# Retention of Antibacterial Activity in Geranium Plasma Polymer Thin Films

**DOI:** 10.3390/nano7090270

**Published:** 2017-09-13

**Authors:** Ahmed Al-Jumaili, Kateryna Bazaka, Mohan V. Jacob

**Affiliations:** 1 Electronics Materials Lab, College of Science and Engineering, James Cook University, Townsville, QLD 4811, Australia; Ahmed.Aljumaili@my.jcu.edu.au (A.A.-J.); kateryna.bazaka@qut.edu.au (K.B.); 2 School of Chemistry, Physics, Mechanical Engineering, Queensland University of Technology, Brisbane, QLD 4000, Australia

**Keywords:** antibacterial coatings, essential oils, geranium oil-derived polymer, plasma polymerisation

## Abstract

Bacterial colonisation of biomedical devices demands novel antibacterial coatings. Plasma-enabled treatment is an established technique for selective modification of physicochemical characteristics of the surface and deposition of polymer thin films. We investigated the retention of inherent antibacterial activity in geranium based plasma polymer thin films. Attachment and biofilm formation by *Staphylococcus aureus*, *Pseudomonas aeruginosa*, and *Escherichia coli* was significantly reduced on the surfaces of samples fabricated at 10 W radio frequency (RF) power, compared to that of control or films fabricated at higher input power. This was attributed to lower contact angle and retention of original chemical functionality in the polymer films fabricated under low input power conditions. The topography of all surfaces was uniform and smooth, with surface roughness of 0.18 and 0.69 nm for films fabricated at 10 W and 100 W, respectively. Hardness and elastic modules of films increased with input power. Independent of input power, films were optically transparent within the visible wavelength range, with the main absorption at ~290 nm and optical band gap of ~3.6 eV. These results suggest that geranium extract-derived polymers may potentially be used as antibacterial coatings for contact lenses.

## 1. Introduction

Medical devices are a critical part of the current healthcare system. However, their usage has led to the emergence of device-associated infections. Contamination of medical devices by microorganisms is associated with substantial morbidity, as well as substantial healthcare costs [1,2]. Abiotic surfaces are vulnerable to microbial attachment and growth, and eventually, biofilm formation; as such, these surfaces may act as a reservoir of chronic infection [3]. Indeed, 64% of hospital-acquired infections worldwide are attributed to attachment of viable bacteria to medical devices and implants [4], and a reported 80% of the global impact of surgical site infections involve microbial biofilms [5]. When protected by the biofilm, bacterial cells are significantly less susceptible to antibiotics and host immune responses than planktonic bacteria of the same strain, and as such, biofilms are more difficult to clear [6]. Emergence of microbial strains resistant to one or more of the available synthetic antibiotics creates further challenges for the treatment of implant-associated infections. Significant examples include methicillin-resistant *Staphylococcus aureus*, NDM-1 producing *Klebsiella pneumoniae*, vancomycin-resistant *Enterococcus*, and multidrug-resistant *Mycobacterium tuberculosis* [7].

To substantially alleviate pathogenic infections, the development of effective self-disinfecting surface coatings for medical devices is vital. Several surfaces have been adapted to inhibit and/or reduce microbial adhesion and proliferation via antibiofouling and/or bactericidal activity, depending on the effect the surface exerts on the microorganism [8]. Antibiofouling materials may resist the initial attachment of microorganisms, due to the existence of unfavourable surface micro-features and/or surface chemistry. On the other hand, bactericidal surfaces may impede colonisation by killing the microorganisms on contact, via surface-immobilised antimicrobial macromolecules, or by releasing biocidal molecules, e.g., polymeric biocides [9]. However, self-disinfecting surfaces often suffer from significant drawbacks, such as uncontrolled material degradation, premature mechanical failure, and limited biocompatibility [10].

In recent years, there has been an increasing interest in the use of plant-derived compounds as natural antimicrobials [11,12]. Essential oils and plant extracts are rich sources of biologically-active compounds with mechanisms that are distinct from those of currently used synthetic antibiotics, which should limit the emergence of bacterial resistance [13]. Among these natural antimicrobial agents, geranium (*Pelargonium graveolens*) essential oil exhibits strong activity against a broad spectrum of bacterial strains [14,15]. When used in solution or as an aerosol, geranium oil was effective against clinically-significant human pathogens, such as gram-positive *S. aureus* and *Enterococcus faecalis*, and gram-negative *P. aeruginosa*, *Proteus mirabilis*, and *Escherichia coli*, and the fungi *Candida albicans* [16,17]. However, until recently, essential oils have rarely been used for fabrication of biologically-active coatings, due to a complex, multi-component nature of essential oils and extracts, which may vary with season and geographic location [18]. Recent advances in cold plasma polymerisation have enabled the conversion of essential oils and their derivatives into thin, highly-adherent and defect-free optically-transparent coatings, whose biological activity and degradation kinetics can be controlled by controlling the chemical structure of the precursor and the plasma processing conditions [19,20,21].

This paper investigates the synthesis, material properties, and antibacterial activity of geranium oil-based polymers fabricated using plasma polymerisation. To our knowledge, this is the first report on the use of polymers derived from geranium oil as an antibacterial coating.

## 2. Methods

### 2.1. Materials

A precursor material was selected because of its strong antibacterial activity against gram-negative and gram-positive bacteria, and compatibility with the plasma polymerisation process [14,22]. The geranium essential oil was procured from Australian Botanical Products (ABP, Victoria, Australia), and was used without further modification. According to the manufacturer, the main compounds include citronellol (32%), geraniol (15%), linalool (6%), isomenthone (6%), geranyl formate (2.5%), tiglate (2%), citronellyl formate (6%), guaia-6,9-diene, and 10-epi-γ eudesmol (5%). Citronellol (C_10_H_20_O) and geraniol (C_10_H_18_) are aromatic acyclic monoterpene alcohols, and are considered to be very potent bactericides responsible for the biological action of geranium oil [23,24,25]. From a processing point of view, geranium oil is compatible with plasma polymerisation (plasma-enhanced chemical vapour deposition), as this oil is highly volatile at room temperature, and so no external heating nor carrier gas is required to deliver the precursor units to the site of deposition. Geranium oil has a density of 1.044 g/mL at 25 °C, a boiling point of 250–258 °C, and refractive index of 1.53 at 20 °C.

### 2.2. Polymer Synthesis

Microscope glass slides (76 mm × 26 mm) and cover glass No.5 (19 mm) were cleaned with commercial decon, washed extensively with distilled water, then sonicated in distilled water for 20 min. Thereafter, substrates were rinsed with acetone, subjected to propan-2-ol bath for 15 min, and finally dried by air. Plasma synthesis was carried out in a custom-made cylindrical glass chamber (*l*: 90 cm, *d*: 5 cm). The reactor chamber was evacuated to pressure of 0.2 mbar using a double stage rotary pump (JVAC–DD150, Victoria, Australia). Radio frequency (RF) generator model ACG-3B (MKS Instruments, Andover, MA, USA) was run in the continuous mode at 13.56 MHz to provide input power through a matching network. Two external parallel copper rings were utilised as electrodes, separated by a distance of 9 cm. Substrates were placed at equal distance from both electrodes. The distance between the substrate and the monomer inlet was 20 cm. Deposition times ranged from 30 min to 120 min, yielding films with thicknesses ranging from ~450 nm to 1500 nm. In each experiment, the monomer vial was loaded with 0.5 g (~12 drops) of geranium oil. The monomer flow rate (*F*) was calculated using the relation 1, derived from the ideal gas equation [26]:

(1) $$F=\frac{dp}{dt}\times 16172\;\frac{V}{T}$$

where *p* is the pressure inside the chamber (mbar), and *t* is time (s), *V* is the volume of the chamber (L), and *T* is the ambient temperature (K).

Initially, the chamber was evacuated to 0.2 mbar, then the precursor gas was released into the chamber until the pressure reached a stable value, at which point the outlet valve was closed, and the pressure was measured every 5 s for 1 min. It was estimated that the geranium flow rate during the polymerisation process was 16.22 cm^3^/min.

## 3. Polymer Characterisation

### 3.1. Chemical Properties

Perkin Elmer Spectrum 100 Fourier transform infrared (FTIR) spectrometer (Perkinelmer Inc., Boston, MA, USA) was employed in the transmission mode to identify components and chemical properties of fabricated films. Films were deposited on potassium bromide pellets (KBr), and the spectra were acquired from 4000 to 500 cm^−1^ with resolution of 4 cm^−1^ averaged across 32 scans.

### 3.2. Optical Properties

Optical constants and film thickness were identified using Variable Angle Spectroscopic Ellipsometry (JA Woollam-M2000 D, Lincoln, NE, USA). Ellipsometry measures a change in polarisation as light is reflected from the surface of a film. The polarisation change is represented as an amplitude ratio (*Ψ*), and the phase difference (*Δ*). The ellipsometric parameters *Ψ* and *Δ* were acquired at three different angles (55°, 60° and 65°, in addition to transmission data) to measure the refractive index (*n*), extinction co-efficient (*k*), and film thickness at wavelength range of 200–1000 nm. The software package (WVASE32, Lincoln, NE, USA) was used for modelling. First, to estimate film thickness, a three-layer model consisting of a previously modelled substrate layer, a Cauchy layer (to represent the film), and a surface roughness layer were applied to the data within the 400–1000 nm region where the film is optically transparent. After point-by-point approximation, optical constants were obtained by converting the Cauchy layer to GenOsc layer and applying Gaussian oscillator to obtain the best fit to the data. UV–vis data were collected using an Avantes spectrophotometer (Avaspec-2048, Apeldoorn, The Netherlands) fitted with an Avalight-DHc light source. Tauc equation was applied to calculate the optical band gap.

### 3.3. Surface Topography and Mechanical Properties

Surface morphology was examined using a low-noise scanning and high-resolution atomic force microscope AFM ( NT-MDT NTEGRA, Moscow, Russian) with a scanning area of 10 µm × 10 µm and 3 µm × 3 µm. AFM was operated in the tapping mode, where the cantilever oscillated directly above the surface to acquire data. This mode was preferable, as it decreased the inelastic deformations of the investigated surface, as well as reduced the effective forces applied to the sample. All measurements were done under ambient conditions. Software Nova (Version 1.0.26, Moscow, Russian) was used to analyse the data with the fitting correction value (polynomial order of 4).

**Figure 1:**
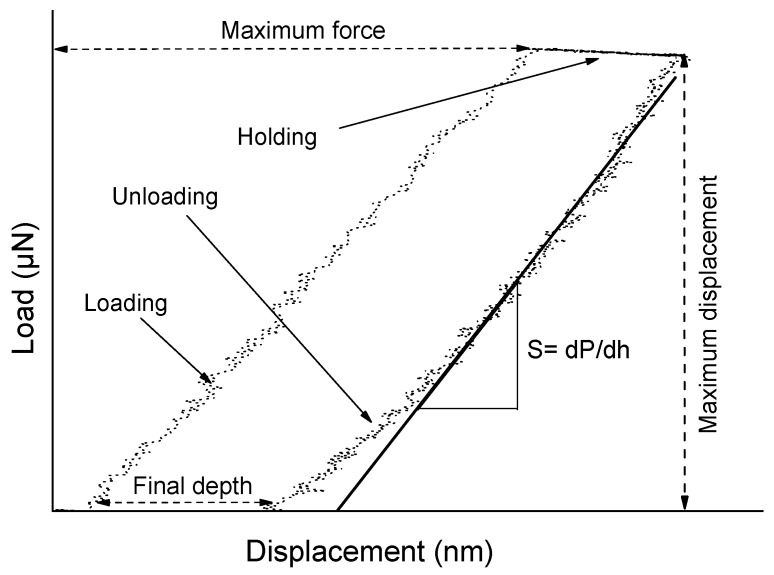
Schematic of the nano-indentation test for hardness and the modulus measurement.

A Berkovich Triboscope indenter (Hysitron, Minneapolis, MN, USA) was interfaced with the AFM Tribo head for the determination of mechanical properties. Berkovich indenter has the geometry of a three-sided pyramid (70.3° equivalent semi-opening angle). To obtain accurate data, the cantilever sensitivity was calibrated prior to measurements using fused silica, then, drift correction was applied. Typical loads ranged from 300 to 1000 µN with a fixed loading time of 3 s, holding time of 3 s, and unloading time of 5 s. Figure 1 shows loading (unloading) versus indenter displacement, illustrating the elastic/plastic response of geranium oil-derived films.

### 3.4. Contact Angle, Surface Tension, and Solubility

Contact angle measurements were used to determine the hydrophobicity of geranium oil-derived thin films deposited on glass. Sessile drop contact angle was measured using goniometer (KSV CAM 101, Helsinki, Finland) and three liquids, namely distilled water, diiodomethane (DIM) and glycerol. Young–Laplace fitting was performed to estimate the contact angle, as a minimum of five measurements per sample.

The calculated values of the contact angle were used to estimate surface tension (also known as surface energy) parameters and solubility of geranium oil-derived polymer films employing van Oss, Chaudhury, and Good (VCG) method [27]:

(2) $$\left(1+cos\theta \right)\gamma_{L}=2\;\sqrt{\gamma_{S}^{LW}+\gamma_{L}^{LW}}+\sqrt{\gamma_{S}^{+}+\gamma_{L}^{-}}+\sqrt{\gamma_{S}^{\_}+\gamma_{L}^{+}}$$

where *θ* is the contact angle, $\gamma_{L}$ the surface tension of the liquid in contact with the solid (mJ/m^2^), $\gamma_{L}^{LW}$ the apolar component of the surface tension of the liquid (mJ/m^2^), $\gamma_{S}^{LW}$ the apolar component of the surface energy of the solid (mJ/m^2^), $\gamma_{S}^{+}$ the electron-acceptor parameter of the solid (mJ/m^2^), $\gamma_{S}^{\_}$ the electron-donor parameter of the solid (mJ/m^2^), $\gamma_{L}^{+}$ the electron-acceptor parameter of the liquid (mJ/m^2^), and $\gamma_{L}^{-}$ the electron-donor parameter of the liquid (mJ/m^2^).

The VCG method requires a minimum of three liquids, an apolar liquid such as DIM (where γ^+^ = γ^−^ = 0), as well as two polar liquids, like water and glycerol. The surface tension parameters of the used liquids are commonly presented in the literature [28], and are summarised in Table 1.

Once the surface tension values for the solid and liquid are determined, the interfacial tension $\gamma_{SL}$ can be calculated from the individual surface tension parameters as:

(3) $$\gamma_{SL}={\left(\sqrt{\gamma_{S}^{LW}}-\sqrt{\gamma_{L}^{LW}}\right)}^{2}+2\;\left(\sqrt{\gamma_{S}^{+}\gamma_{S}^{-}}+\sqrt{\gamma_{L}^{+}\gamma_{L}^{-}}-\sqrt{\gamma_{S}^{+}\gamma_{L}^{-}}-\sqrt{\gamma_{S}^{-}\gamma_{L}^{+}}\right)$$

The solubility of the geranium oil-based polymer films (Δ*G*) in different solvents is then estimated from the values of the interfacial tension as:

(4) $$\Delta G_{121}=-2\gamma_{SL}$$

### 3.5. Bacterial Studies

#### 3.5.1. Cell Cultures

Among clinically-significant pathogenic bacteria, infections due to the Staphylococci, particularly gram-positive *S. aureus*, and gram-negative *P. aeruginosa* and *E. coli*, are most frequently associated with the use of implants [8,29]. Given their significance, in this study, *S. aureus* CIP 65.8, *P. aeruginosa* ATCC 9025, and *E. coli* K12 were acquired from the American Type Culture Collection (ATCC, Manassas, VA, USA) and Culture Collection of the Institut Pasteur (CIP, Paris, France). For each experiment, a fresh suspension was prepared by first refreshing the frozen stock culture on nutrient agar (Oxoid), then growing them overnight in 100 mL of nutrient broth at 37 °C, while shaking at 100 rpm. Cells were harvested at the logarithmic stage of growth, and their density adjusted to OD_600_ = 0.3 to ensure uniform starting culture. A haemocytometer was used to quantify cell numbers in suspension prior to seeding onto polymer surfaces.

#### 3.5.2. Incubation

Sterile polymer-coated glass slides and uncoated glass slides (as control) were placed into 24 well plates, and an aliquot of 100 μL of bacterial suspension was carefully placed on the sample surface. Samples were then allowed to incubate at 37 °C and 5% CO_2_ for 18 h. The experiment was run in triplicate. After incubation, the media was aspirated, and the unattached cells were removed by gently rinsing the surfaces of the samples with copious amounts of double-distilled water. Samples were then allowed to dry at 22 °C for 30 min at 55% humidity to maintain cells in a semi-hydrated state for microscopy.

#### 3.5.3. Visualisation

The retained bacteria were visualised by scanning electron microscopy. Prior to imaging, samples were coated with a thin layer of gold using Sputter Coater (Leica EM-SCD005, Wetzlar, Germany). High-resolution images of the attached cells were obtained using the Scanning Electron Microscope (JEOL 7001F, Tokyo, Japan) at 1000, 5000, and 20,000× magnifications.

To differentiate viable cells among the attached bacteria and quantify the amount of extracellular polysaccharides produced by the attached cells, SYTO^®^ 17 Red (Molecular Probes™/Invitrogen, Thermo Fisher, Carlsbad, MA, USA) and Alexa Fluor^®^ 488 (Molecular Probes™/Invitrogen, Thermo Fisher Scientific, Carlsbad, MA, USA) stains were used to stain the bacterial cell red and extracellular polymeric substances (EPS) green, respectively. The dyes were applied following the protocol outlined in [30]. The images were obtained using a confocal scanning laser microscope (CSLM) (Nikon A1R Confocal Microscope, New York, NY, USA). The CSLM images were processed to construct 3D images, and to quantitatively describe the images in terms of the overall volume of the biofilm per unit area of substrate (termed “biovolume”, and inclusive of both cells and EPS), and the average biofilm thickness.

## 4. Results and Discussion

### 4.1. Polymer Synthesis

The polymer thin films were successfully fabricated on substrates, including microscope glass slides, cover glass, and KBr disks, at input powers of 10, 25, 50, 75, and 100 W. Spectroscopic ellipsometry was used to estimate the polymer thicknesses. The thickness of the polymer increased linearly with time, yielding ~10.6 nm/min at 10 W. Also, the thickness of the polymer was found to be increasing with an increase in the input power.

### 4.2. Chemical Properties

Geranium essential oil is a complex, multi-component mixture of monoterpenic alcohols (geraniol, citronellol, linalool, etc.), esters (citronellyl tiglate, citronellyl formate, geranyl tiglate, etc.), monoterpenic ketones (isomenthone and menthone), monoterpenic aldehydes (geranial and neral), sesquiterpenic hydrocarbons (guaia-6,9-diene, etc.), sesquiterpenic alcohol (10-epi-γ-eudesmol), and several aromatic and oxide components [31]. Citronellol and geraniol are considered the key components, while linalool is present in a much smaller portion (for more detail on oil composition see Section 2: Materials).

Principally, the organic molecules are held together via covalent bonds, which are relatively strong, while creating a solid material via fairly weak van der Waals forces [32]. During plasma polymerisation, plasma-generated electrons gain sufficient energy to break chains of the precursor molecules, creating a rich assortment of highly reactive chemical species. The degree of fragmentation is directly related to the amount of applied energy. Thus-created precursor fragments then undergo recombination in the gas phase and on the surface of the substrate, giving rise to a highly crosslinked polymer, with a structure that is irregular and unlike that of a conventional polymer.

**Figure 2:**
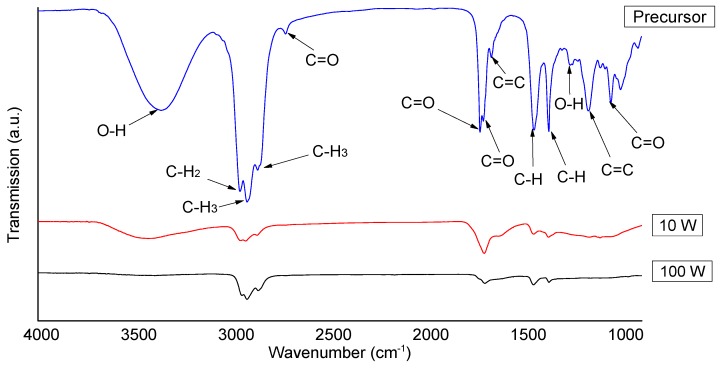
FTIR spectra of geranium essential oil (precursor) and geranium oil-based polymer fabricated at 10 W and 100W.

Figure 2 shows the FTIR spectra obtained for geranium oil (precursor) and geranium oil-based polymer thin films. Table 2 summarises the key bands and corresponding bond vibrations. In the precursor spectrum, a very broad and strong peak of around 3367 cm^−1^ was related to the stretch vibrations of (O–H) bonds of the alcohol. In the film spectrum, this band reduced in intensity and appeared at 3436 cm^−1^. The very strong peaks formed at 2962 and 2872 cm^−1^ can be attributed to the asymmetric stretching vibration modes (CH_2_ and CH_3_, respectively) of the methyl group. In the polymer, these peaks also decreased in intensity and occurred at 2961 and 2875 cm^−1^, respectively. A very strong stretch vibration of the CH_3_ bond appeared at 2926 cm^−1^, and related to the methylene group, shifted slightly in the polymer spectrum towards a higher wave number at about 2933 cm^−1^. A very weak peak at 2728 cm^−1^, related to C=O bond stretching, vanished completely, and was not observed in the polymer spectrum. Strong peaks at 1730 and 1713 cm^−1^, possibly linked to the symmetric starching vibrations of C=O bonds, and a medium peak at 1671 cm^-1^, probably related to the vibration of (C=C) were observed in the precursor spectrum. These bands merged, decreased in strength, and appeared as a broad band in the polymer spectrum at about 1708 cm^−1^. Sharp peaks at 1452 and 1377 cm^−1^ were attributed to the asymmetric and symmetric bending vibrations of C–H bonds, respectively. These vibrations were retained in reduced form and observed in the polymer at 1453 and 1376 cm^−1^, respectively. Furthermore, peaks observed in the fingerprint region of the precursor at 1267, 1174, 1058, and 1008 cm^−1^, combined and radically reduced in the polymer spectrum, and were detected as very weak and united broad bands.

The reduction in the peak intensities and disappearance of some peaks in the polymer can be interpreted as the precursor molecules being partially dissociated as a result of being subjected to the RF plasma field [33]. However, the absorption band of C=O asymmetric stretching observed in the 1800−1600 cm^−1^ region was possibly due to the post oxidation of the trapped free radicals, confined during film formation [34]. The emergence of a strong methyl peak at 1452 cm^−1^, and relatively weaker methylene band at 1377 cm^−1^, confirms that geranium oil-derived films comprise a large quantity of short unsystematically-branched chains, rather than long linear backbone structures, a structure typically associated with plasma polymer [26].

Comparison of the resulting films at different deposition powers (10 W and 100 W) revealed that with increasing input power, the crosslinking of the fabricated polymers increases. This is possibly owing to the higher fragmentation rate that occurs in higher density plasmas, due to an increase in inelastic collisions among high energetic electrons and precursor species, which could cause the overlapping of electronic orbitals [34].

### 4.3. Optical Properties

Determination of material optical properties, including transparency, the spectral dependence of the refractive index and extinction coefficient, and optical band gap energy, are essential in optics-related industries [35,36]. Independent of applied power, geranium oil-derived polymer films were revealed to be optically transparent within the visible wavelength range.

**Figure 3:**
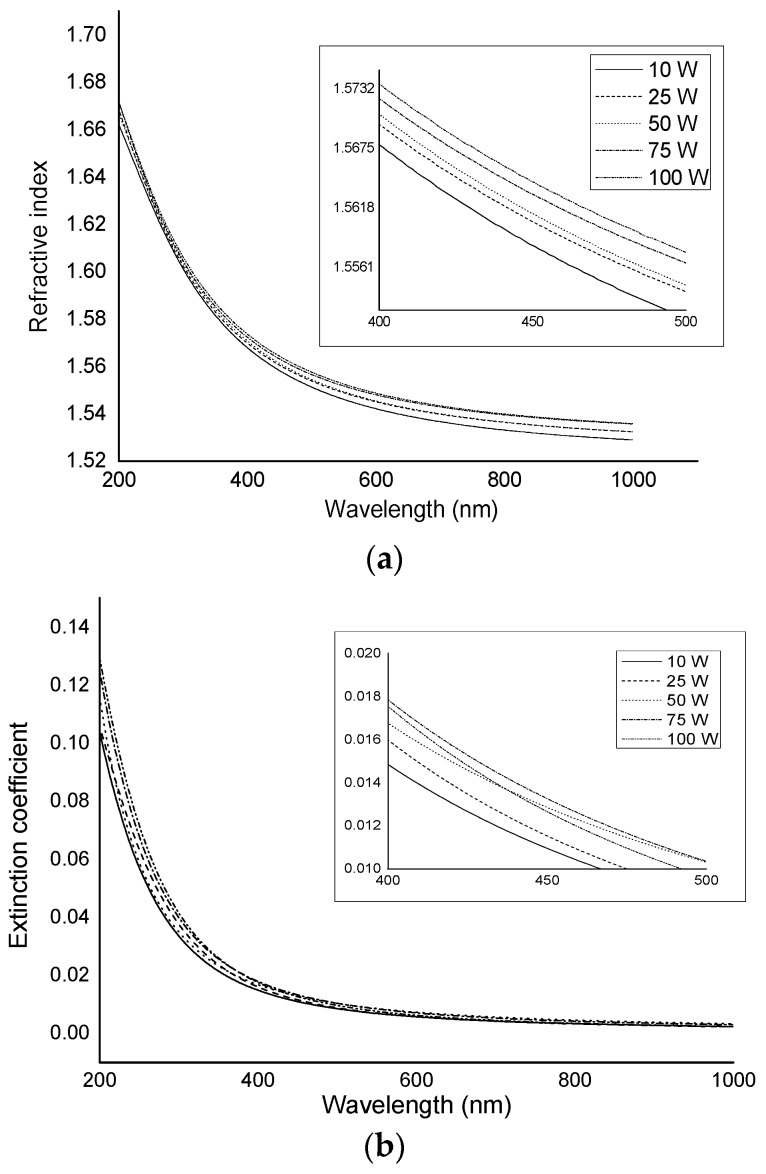
Optical constants of geranium oil-derived polymer films fabricated at various deposition powers; (**a**) Refractive index; (**b**) Extinction coefficient.

Refractive index (*n*), a measure that describes how light propagates through the medium, was found to be not significantly dependent on the RF power, with all curves characterised by a similar shape (Figure 3). At a short wavelength of 200 nm, the variation in the refractive index between polymers fabricated at applied powers of 10 W and 100 W was approximately ~0.0097. At wavelengths above 900 nm, the variation in the refractive index was ~0.0069. This result agrees with previous studies that showed a variation of less than 1% for thin films derived from other essential oil precursors, e.g., γ-terpinene and linalyl acetate [37,38]. In contrast, Cho et al. found that the refractive index of ethyl-cyclohexane films increased when the RF power was increased [39].

The extinction coefficient (*k*), a measure of how a medium absorbs light at a specified wavelength, also showed very little dependence on the applied power, especially in the high wavelength region (above 900 nm), as the variation was 0.0013. At a short wavelength of 200 nm, the variation in the refractive index for polymers fabricated at applied powers of 10 W and 100 W was ~0.0268. As the films have optical constants similar to glass and good transparency in the visible region, it is suggested they are suitable candidates for integration into protective coatings in optical and biomedical devices, such as lenses [38,40].

**Figure 4:**
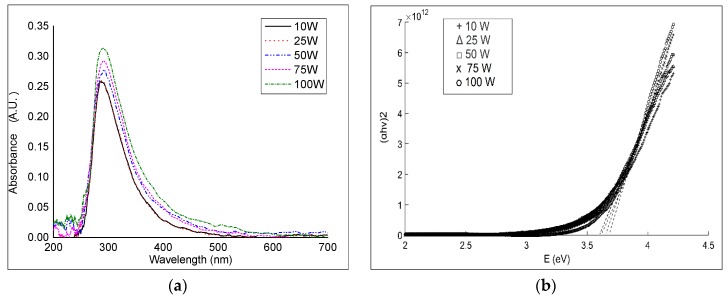
(**a**) UV–vis absorption spectrum of geranium oil-derived films; (**b**) The optical energy gap of geranium oil-derived films fabricated at various radio frequency (RF) powers.

Figure 4 shows UV–vis spectra of geranium oil-derived polymer thin films deposited on glass with increasing RF powers, from 10 to 100 W. The maximum absorption peak was observed at approximately 290 nm, which possibly relates to π–π* transitions [41]. The absorption peak width increased slightly with an increase in the RF power. This could be associated with an increase in the length of the conjugated π-system, which usually shifts the absorption maximum peak to a longer wavelength [42]. However, no significant shift in location or variation in magnitude of the absorbance peak was detected with respect to RF power. This was also observed in films fabricated from linalyl acetate by plasma polymerisation [38]. The findings show that the absorption spectra are repeatable under similar deposition conditions (RF power, pressure, temperature, etc.). The optical absorbance results strongly suggest that geranium films can be used as encapsulating (protective) layers for organic electronics to extend the lifetime and preserve efficiency of oxygen- and water-sensitive organic materials [40].

The energy gap of geranium oil-derived polymer films was determined using the optical absorption coefficient data acquired from UV–vis spectroscopy measurements. The optical energy gap was calculated using Tauc relation *αE* = *A*(*E* − *E*_g_)^1/*n*^, where *E* is the photon energy, *A* is a constant, and *n*(1/*r*) is related to the density-of-states distribution in the transport gap, and equal to 1/2, 3/2, 2, or 3 for direct or indirect transitions [43]. The MATLAB program was used to convert UV–vis spectroscopy data to a Tauc plot, determine the linear portion of the high energy region, and extrapolate the line until it intersected with *x*-axis to estimate the optical band gap (Figure 4). The best fit was observed at *n* = 1/2, which is related to directly allowed transitions.

A very small decrease of optical band gap with an increase in the input power was observed in the films. Samples fabricated at 10, 25, 50, 75, and 100 W had *E*_g_ ≈ 3.67, 3.65, 3.60, 3.61, and 3.60 eV, respectively. The optical properties of plasma polymerisation films significantly relied on the structure of the p-conjugated chains in both the ground and the excited levels, and on the inter-chain orientation [44]. The narrowing of *E*_g_ is possibly owed to dangling bonds created in the polymer structure during the fabrication. At low RF power, there was low concentration of dangling bonds because of their saturation with hydrogen atoms, while higher RF power increased the fragmentation rate in the plasma field that accelerated deposition of chains with unsaturated bonds [45]. The unsaturated bonds were expected to be reason for the foundation of structural defects and/or created some intermediate energy levels due to structural reorganisations enhancing the density of localised states in the band structure that end in low values of *E*_g_.

### 4.4. Surface Topography

Topological and biochemical characteristics of a surface in part determine the rate of microbial adhesion. Bacterial growth and proliferation are highly associated with the size of the micro/nano-features of the surface. There is an ongoing debate among scientists regarding preferential attachment of bacteria to rougher surfaces. Proponents of the theory attribute increased cell adhesion to three main factors, namely higher surface area available for attachment, protection from shear forces, and chemical changes in cells that cause preferential physicochemical interactions [46]. At the microscale, where the cell is much smaller than surface features, the roughness would indeed provide “hiding places”. While at nanoscale, where the features are smaller than the size of the cell, rougher surfaces would provide less points of attachment than smooth surfaces. Also, the distribution of the features on the surface is important. It has been reported that *Pseudomonas aeruginosa* and *S*. *aureus* cells preferentially attached to surfaces constructed of regularly spaced pits of 1 µm and 2 µm in size, but not those constructed of irregularly spaced pits of 0.2 µm and 0.5 µm [47].

**Figure 5:**
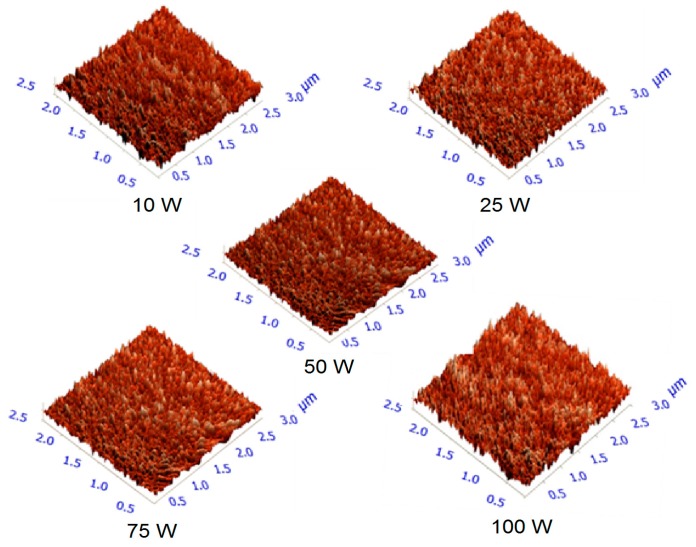
Typical three-dimensional atomic force microscope images of 3 µm × 3 µm scanning area of geranium oil-derived film surfaces fabricated at various RF power.

In this study, AFM images were obtained to determine information regarding the surface properties. Several parameters were used to describe the surfaces, including maximum height (*S*_max_), average roughness (*S*_a_), root mean square (*S*_q_), skewness (*S*_sk_), and kurtosis (*S*_ka_). *S*_max_, *S*_a_, and *S*_q_ were used to estimate the topographical characteristics of geranium oil-derived polymer films, while *S*_sk_ and *S*_ka_ were referred to when describing the surface regularity. Representative images of the surfaces of geranium oil-derived films are shown in Figure 5, and roughness parameters for the films are summarised in Table 3.

The maximum peak height of 8.30 nm was observed on the surface of the sample fabricated at the highest deposition power (100 W) with a scanning area of 3 µm × 3 µm. *S*_a_ and *S*_q_ increased slightly as a result of an increase in the input RF power. At powers of 10, 25, and 50 W, *S*_a_ values were 0.18, 0.21, and 0.29 nm, respectively, with higher *S*_a_ values reported for the samples deposited at 75 and 100 W, at 0.63 and 0.69 nm, respectively. The increase in the roughness may be related to more energetic ions at higher RF power causing more surface bombardment and etching [48]. Irrespective of fabrication power, the average roughness values remained below 0.7 nm, confirming the smooth and uniform nature of plasma polymers from geranium oil.

Surface skewness measures the symmetry of the deviations of a surface profile about the mean line. Its value can be positive or negative, and it is sensitive to the irregularity of deep valleys or high peaks [49]. The skewness parameter can be employed to distinguish between surfaces with similar root mean square roughness or arithmetic average height values, but different shapes [50]. The maximum value of skewness for geranium oil-derived plasma polymer films was found to be 0.75 nm on the sample deposited at 75 W (10 µm × 10 µm). Another parameter frequently used to describe surfaces, surface kurtosis (*S*_ka_), describes the distribution of the protrusions with respect to the mean line. For a mesokurtic distribution that is similar to or identical to normal distribution, the kurtosis is zero. Distributions with positive kurtosis are leptokurtic, and are characterised by high peaks, whereas platykurtic distributions have negative kurtosis, and are characterised by flat-topped curves [51]. All investigated samples exhibited *S*_sk_ and *S*_ka_ values falling fairly close to 0 and 3, respectively, suggesting surfaces with a symmetrical distribution of peaks and valleys [29]. It has been observed that skewness and kurtosis parameters of geranium oil-derived films are independent of the RF deposition power.

Independent of deposition power, the topographical features appeared to be uniform, smooth, and pinhole free. The uniformity indicates that polymerisation reactions occurred essentially on the surface of the substrate, instead of in the gas phase. The variances in the sample profiles, and both scanning areas, were not statistically significant. Besides possessing considerable smooth surfaces, films fabricated by the plasma polymerisation process reveal high spatial uniformity and good adhesion to the substrate [52]. AFM results also revealed that the films’ entropy, or the level of disorder or randomness in a system, increased as a result of fabrication power. The entropy increases are possibly related to the surface flatness decreases [53].

### 4.5. Mechanical Properties

**Figure 6:**
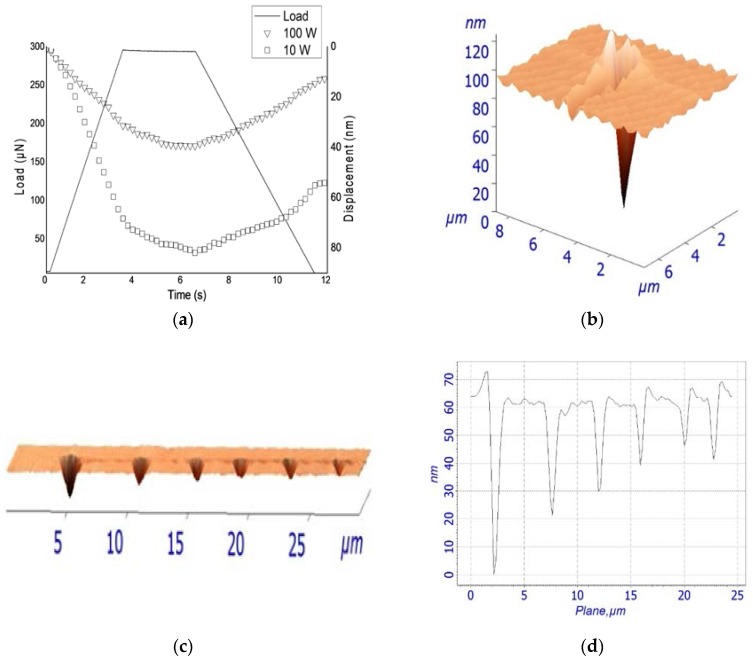
(**a**) Load displacement versus time curve of geranium oil-derived film fabricated at 10 W and 100 W. (**b**) Pile up and sink in phenomena in the film. (**c**) AFM image of plastic impressions left behind in geranium oil-derived thin polymer fabricated at 50 W after indentations. (**d**) Profile plane of the investigated surface.

In order to determine the mechanical properties of geranium oil-derived thin films, a nano-indentation test was performed. The AFM was activated in the force mode that brought the tip into contact with the tested film, pushed to a maximum load, held for a period of time, and then withdrawn. While the indenter was being pushed into the polymer, the load and displacement were identified continuously, drawing a load versus displacement curve, as presented in Figure 6A. For very thin polymer films, the quality of the data is limited by a number of factors. It has been discussed that creep effects on elastic modulus at higher loads are significant, even though this influence could be a result of higher unloading stiffness [54]. Thermal drift, caused by inconsistency in ambient temperature, complicates nano-indentation measurements. Peaks and valleys can affect how the indenter contacts the sample. Furthermore, the pile up and sink in phenomena are related to plastic deformation in the films, as seen in Figure 6B. During the holding time, it is possible that the tip would continue to move into the surface of the sample as a result of the viscous creep. Therefore, six indents were randomly located on the films to reduce the inaccuracy due to creep or roughness effects. The loading forces of 300, 400, 500, 600, 700, and 1000 µN were applied, with indentations separated by at least 5 µm, as presented in Figure 6C,D. In order to minimise the effect of the substrate on the measurement, films with thickness of >1500 nm were tested to keep indentation depths within 10% of the film thickness, thus collecting data within the plastic response of the film only.

The hardness of geranium oil-derived thin films increased with the increase of power deposition, as shown in Table 4. This could be associated with the increase in crosslinking in the polymers deposited at higher RF power, which potentially results in an increase in resistance against deformation [55,56]. Similarly, an increase in elastic modulus and a decrease of contact depth with increasing RF power, have been observed. This may be attributed to the transition from spherical contact to conical contact that occurs for the Berkovich indenter used in the investigation at h_c_ = indenter radius/4 [37]. Liu et al. argued that the Berkovich indenter (complex shape) has higher hardness and lower elastic modulus compared to the conical indenter [57]. It is not uncommon to observe the stress relaxation at the maximum load when unloading just takes place, since the strain beneath the indenter is relatively large, and the strain rate is also very large at this point [58]. Besides, the sudden withdrawal of the indenter leads to oscillations, which may affect the measurements.

The elastic modulus and the hardness were calculated using the Oliver–Pharr nonlinear curve, which is based on the equation *P* = *A*(*h* − *h_f_*)*^m^*, where *A* and *m* are power law fitting parameters, *h* is the depth variable, and *h_f_* is the final depth. In light of what has been written on measurement limitations (above), the hardness and elastic modules values presented in Table 4 may quantitatively differ from the actual properties of geranium oil-derived thin polymer films.

### 4.6. Contact Angle and Wettability

The wettability of plasma polymerised films was assessed using the sessile liquid drops system. A liquid drop was gently placed on a horizontal solid substrate, where the drop formed the shape of a sphere section due to its interaction with the surface. The angle at the triple-phase contact line between the sphere and the surface was measured to determine the wettability of the surface. Theoretically, the adhesive forces between a liquid drop and a substrate are a local reaction, which is influenced by the interactions of the actual drop and the surrounding vapour with the substrate, thereby necessitating independence of the drop volume [59]. However, it has been argued that larger drops are more subject to errors as a result of gravity effects, causing sagging near the contact line, and deformations to the drop [60]. In contrast, smaller drops can cause errors due to evaporation and the interference of contact line tension, triggering deviations in measurements [61]. Further, the atmospheric conditions play a substantial role in the control of contact angles, where the partial pressure of oxygen influences the equilibrium and uniformity of the formed drop [62]. Besides, the homogeneity of the chemical composition of the surface may affect droplet symmetry [63,64]. The topography is a parameter that has to be considered, as in some cases, contact angles can be increased owing to the roughness [65]. However, contact angle measurements provide details about the character of a top surface in the range of 0.5–1.0 nm [66].

For the above reasons, the size of the droplet was carefully chosen to minimise inaccuracies in the acquired data for the test liquids. In the case of the water, a droplet volume (*V*) of approximately 3 µL was used, yielding dimensions of area (*A*) ≈ 9 mm^2^, height (*H*) ≈ 0.90 mm, and base diameter (2*a*) ≈ 3 mm upon contact with polymer surface. For DIM (CH_2_I_2_), a smaller droplet of *V* ≈ 1.3 µL was used, producing dimensions of *A* ≈ 6.9 mm^2^, *H* ≈ 0.39 mm, and 2*a* ≈ 2.8 mm upon contact with polymer surfaces. For glycerol (C_3_H_8_O_3_), an average droplet volume of *V* ≈ 1.6 µL was used, resulting in contact droplet dimensions of *A* ≈ 5.7 mm^2^, *H* ≈ 0.66 mm, and 2*a* ≈ 2.33 mm. For these dimensions, the relative error was ~1%.

In each experiment, the drop profile was recorded by video camera and solved numerically. Static contact angle data were acquired for each sample on a minimum of five points. Table 5 presents average contact angles of geranium oil-derived polymer films fabricated at various RF power levels. At the higher power of 100 W, water contact angle was 65.6°, while at the lower power of 10 W, the contact angle value was notably lower, at 55.5°. An increase in the contact angle with an increase in the fabrication power may be attributed to a decrease in the oxygen content in the resultant polymer films. This conclusion is supported by FTIR data, which show the hydroxyl group peak reduced in intensity as RF power increased, which in turn led to a decrease in the polarity of the surfaces of films fabricated at higher RF power [67,68].

**Figure 7:**
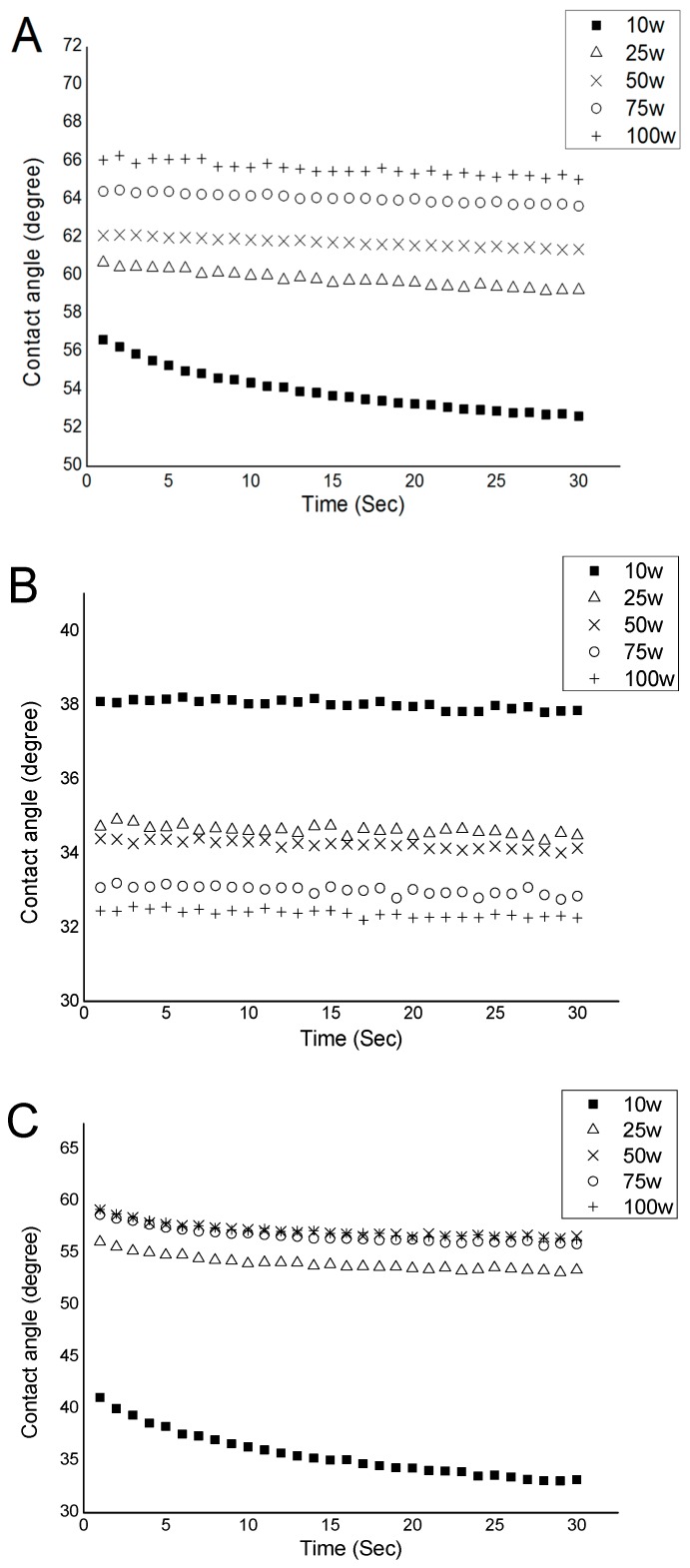
Evolution of contact angle with contact time: (**A**) water, (**B**) DIM, and (**C**) glycerol.

Examination of the evolution of contact angle with time (Figure 7) provides further evidence for the dependence of contact angle on the degree of crosslinking, with a relatively high rate of change in the case of polymers fabricated at lower RF power. This behaviour is indicative of the reorientation of functionalities at the solid–liquid interface in the films fabricated at low power, which is difficult in more crosslinked films synthesised under high RF power conditions [27]. The formation of highly crosslinked structures in films is probably owing to more fragmentation of precursor molecules as the applied power increases [27]. The resultant films become more rigid because of an increase in the bonding interconnection and dense packing. An increase in the water contact angle, with respect to input power of polyterpenol coatings deposited by plasma polymerisation, has also been reported [69].

Geranium oil-derived polymer films revealed contact angles ranging from ~50° to 60°. Moderately hydrophilic surfaces are generally considered well-suited for biological applications, as they facilitate and promote adhesion of multiple cell lines, and hence can be used as coatings to enhance biocompatibility of implantable and extracorporeal biomaterials [68,70]. Once the equilibrium contact angle was reached, the contact angle remained stable, indicating chemical stability of geranium oil-derived films while in contact with water. The contact angle for diiodomethane decreased with increasing RF power, whereas contact angle for glycerol showed similar behaviour with increasing RF power, to that of water.

### 4.7. Surface Tension Parameters and Solubility

There are several approaches to estimate the surface tension through contact angle, such as the methods by Berthelot, by Fowkes, and by van Oss, Chaudhury, and Good (VCG) [27,71,72]. It should be noted that there are unique limitations for using each of these methods. Nevertheless, the VCG three-liquid approach has been widely studied and successfully employed for determining surface tension parameters [73], and thus, was selected to estimate the surface tension values in this study. The VCG method considers the total surface tension γ of a solid surface is a summation of interfacial Lifshitz–van der Waals forces γ*^LW^* (dispersive and polar) and acid–base interactions γ*^AB^* (electron donor–acceptor), where $\gamma^{AB}=2\;\sqrt{\gamma^{+}\gamma^{-}}$ [67].

The acquired contact angle data were used to determine the surface tension parameters summarised in Table 6. The results indicate that an increase in RF power had a slight influence on the total surface tension of the geranium oil-derived polymer films. In particular, samples fabricated at 10 W revealed relatively high surface tension compared to samples fabricated at other RF powers. The dispersive parameter γ*^LW^* showed a consistent decrease with increasing RF power. This may be because the higher power samples contained less polar moieties, such as –OH group [28]. The surface tension parameters calculated from polar liquid data, i.e., water and glycerol contact angles, decreased for increasing RF deposition power, due to an increase in the polarity of these surfaces, while the surface tension parameters estimated from contact angles for apolar liquid (DIM) displayed the opposite trend.

The highest value of γ^+^ for geranium oil-derived polymer films was 5.88 mJ/m^2^, calculated for 10 W samples, and the lowest value was 0.87 mJ/m^2^ for 50 W samples. The maximum value of γ^−^, 29.82 mJ/m^2^, was obtained for films deposited at 10 W, and the lowest value was 15.85 mJ/m^2^, calculated for samples fabricated at 100 W. All samples displayed γ^−^ > γ^+^, indicating that the tested films have monopolar surfaces, of which most are water soluble [67]. To evaluate the minimum value for γ^−^ parameter at which the polymer becomes soluble in the water, the van Oss formula Equation (2) was solved for γ*^LW^*= 42.26 mJ/m^2^. It was found that geranium oil-derived plasma polymer films become water-soluble at γ^−^ > 28.96 mJ/m^2^. Thus, samples fabricated at 10 W are expected to be soluble in water, while samples fabricated at higher RF power (25, 50, 75, and 100 W) are expected to resist solubility, since their γ^−^ < 28.96 mJ/m^2^.

The determination of polymer solubility (ΔG_121_) is vital for biomaterial applications, since the material deals mainly with aqueous media, as well as for solution processing in electronics. Physically, ΔG_121_ represents the free energy change, so when ΔG_121_ >> 0, the material is solvophilic in the used liquid; while for ΔG_121_ << 0, the material is solvophobic in the used liquid; for ΔG_121_ ≈ 0, the material is partially dissolved in the given solvent. Solubility of geranium oil-derived films in different solvents are reported in Table 6. The polymer films were found to be solvophobic for all solvents, except for 10 W sample. The solvophobicity behaviour of geranium oil-derived films increased with input power for water and glycerol (polar solvents), while they decreased slightly for DIM (apolar solvent). This is possibly related to the increase in the polarity of the polymer surfaces, as discussed previously. The solubility results showed that the stability of fabricated polymers is influenced by both fabrication conditions, namely RF power, and the choice of the solvents. However, it is important to note that the solubility values may be quantitatively different when using another approach in the calculations of surface tension.

### 4.8. Cell Attachment

**Figure 8:**
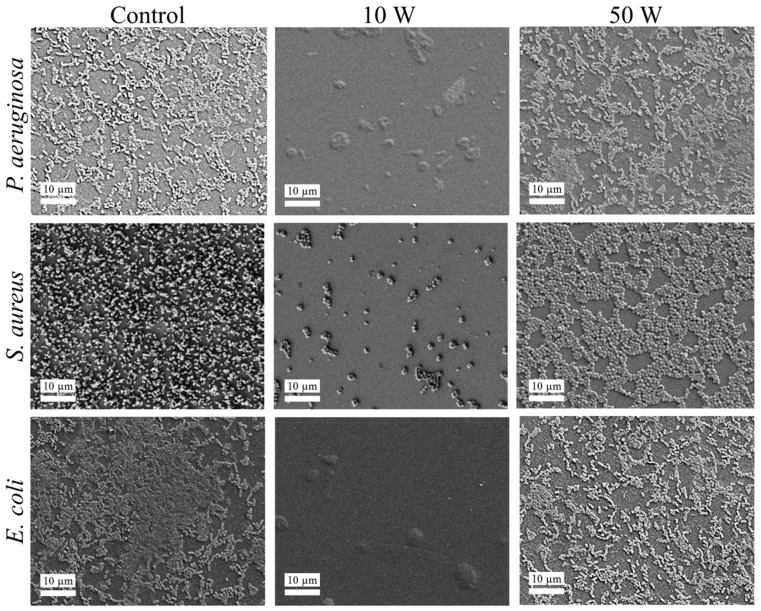
Representative *P. aeruginosa*, *S. aureus*, and *E. coli* attachment patterns on the surfaces of the control glass, and geranium oil-derived polymer film samples fabricated at 10 W and 50 W after 18 h incubation. SEM images represent an overview of the attachment pattern.

The attachment and biofilm formation of *S. aureus*, *P. aeruginosa*, and *E. coli* on the surfaces of geranium oil-derived polymer films after 18 h of incubation were visualised by SEM and CSLM imaging (Figure 8), and quantified (Table 7). Comparison of attachment patterns revealed notable differences for samples fabricated at different RF powers, regardless of the pathogen tested. Films fabricated at 10 W RF power displayed significant antifouling activity, preventing the attachment of bacterial cells, as well as limiting the formation of biofilm. Bacterial cells produced significantly more EPS when attached to glass control or polymer film fabricated at 50 W RF power, forming double-layer morphology, indicative of the biofilm formation. The morphologies of the cells were also different between the surfaces, with smaller cells being present on the surface of the 10 W sample compared to that of cells attached to 50 W sample or glass control. There was no significant difference between the number of cells, biovolume, or biofilm thickness obtained for the 50 W sample and control.

These results are very similar to that reported for polyterpenol thin films, which were synthesised using plasma polymerisation from terpinene-4-ol, a broad-spectrum antimicrobial plant secondary metabolite and a major constituent of *Melaleuca alternifolia* essential oil [69]. There, low input power conditions (i.e., 7–10 W) were also found to favour the partial preservation of biochemical activity of the original monomer unit, and resulted in a substantial antimicrobial and antibiofouling activity of polyterpenol thin films. Similarly to this study, those *S. aureus* cells that managed to attach to the surface of polyterpenol were of smaller size than those attached to control or polyterpenol films fabricated at high input power. At 0.6 μm, they were also smaller than those cells attached to the surfaces of geranium oil films fabricated at the same input power conditions (at 0.8 μm). The biofilm formation was also reduced on polyterpenol samples deposited at 10 W, at an average thickness of 0.30 ± 0.04 µm, and biovolume of 0.09 ± 0.002 µm^3^/µm^2^, which is similar to the respective values of 0.35 ± 0.03 µm and 0.28 ± 0.03 µm^3^/µm^2^ observed on geranium thin films. With a similar level of activity, geranium oil presents a more attractive alternative, considering that it contains multiple constituents, and thus, may potentially target different cellular components and processes within the bacterium, thus contributing to the efficacy of the coating and potentially reducing the likelihood of bacterial resistance.

The nature of cell–surface interactions is dependent on the properties of both the pathogen cell and the surface the pathogen is trying to colonise [74]. It is well-established that the mechanism of bacterial attachment is multi-stage, involving reversible and irreversible components. When bacterial cells are separated from the surface by the distance of more than 50 nm, the interactions between the cells and the surface are nonspecific, and are determined by both the separation distance and the free energy of both entities, in particular, their respective dispersive components. Attractive forces promote cell attachment, whereas repulsive forces impede the ability of the cell to approach the surface and engage in more specific molecular or cellular interactions. Those cells that manage to successfully approach the surface (where the distance is below 5 nm) have the ability to engage in hydrogen bonding, ionic and dipole interactions, and hydrophobic interactions. These interactions are more difficult to break. To assist attachment, bacterial cells can actively express a wide range of surface-bound and free-floating polymeric structures, such as capsules, fimbriae, pili, and slime, which may facilitate cell movement toward the surface and the establishment of specific, irreversible molecular bonds with the colonised surfaces.

*S. aureus* cells are moderately hydrophobic, with a typical water contact angle of ~70°, whereas *P. aeruginosa* and *E. coli* cells are moderately hydrophilic, with a water contact angle of ~45° and ~35°, respectively. The surfaces of the three pathogens are negatively charged, with *E. coli* exhibiting the largest negative zeta potential ζ of −39.5 mV, followed by *S. aureus* (ζ = −33.1 mV), with *P. aeruginosa* cells characterised by the least negative zeta potential of −15.1 mV. Given that geranium oil-derived polymer films tend to donate electrons rather than accept them, some electrostatic repulsion would be expected to take place between the bacterial cells and the negatively charged polymer surface, with the strongest repulsion, and hence, weakest cell attachment, expected for *E. coli* cells. Interestingly, independent of the pathogen species, geranium oil-derived polymer surfaces were susceptible to bacterial colonisation and some biofilm formation. Conversely, polymer samples fabricated at 10 W actively repelled cell attachment irrespective of bacterial species, suggesting a more complex mechanism of antibacterial activity.

Exposure of the precursor to the highly reactive plasma environment initiates a wide range of reactions that include fragmentation, rearrangement, oligomerisation, and polymerisation. The extent of precursor fragmentation is highly dependent on the amount of energy delivered into the plasma chamber, which is in turn, directly related to the applied RF power. The dissociation is initiated by highly energetic electrons, rather than by means of thermal excitation or chemical reaction, giving rise to a unique assortment of chemically reactive species that may not be obtainable under other processing conditions. These reactive species can undergo recombination inside and outside of the plasma region, e.g., at the surface of the substrate, enabling the formation of the polymer thin film on its surface. Given the abundance of chemically-diverse species, and the presence of functional groups typically associated with conventional polymerisation, the polymerisation process follows multiple pathways, including conventional polymerisation, as well as fragment-recombination triggered by the plasma-generated and surface-attached reactive ions, and free radicals. This gives rise to a more complex polymer structure, potentially rich in free radicals trapped in a three dimensional network. The surface topography of the thus-formed polymer is influenced by the intensity of plasma-generated ion bombardment, which is again linked to the applied RF energy.

Considering the intimate link between biological activity of the surface and its surface chemistry and nanoscale topography, it is possible that the combination of these properties in polymer films fabricated at 10 W prevent bacterial fouling. Chemical characterisation showed that these surfaces bared a larger proportion of hydroxyl functional groups compared to the samples fabricated at 50 W. It has previously been shown that *S. aureus* cells preferentially attached to surfaces bearing carboxylic and methyl functional groups than those containing –OH functionality [75]. This is also supported by the thermodynamically predicted preference of hydrophobic cells for hydrophobic substrates.

## 5. Conclusions

We have synthesised and investigated the properties of geranium extract-derived polymer thin films at various fabrication powers. With increasing input power, the crosslinking of the fabricated polymers increased. The refractive index, extinction coefficient, and optical band gap were found to be not significantly dependent on the RF power. AFM images indicated that the topographical features appeared to be uniform, smooth, and pinhole free for all samples, and the surface roughness increased with an increase in the input power. The nano-indentation test revealed that the hardness and elastic modulus of the films increased with RF power deposition. The wettability of the polymer improved with input power and the polymer became more resistant to solubility in water.

Geranium extract-derived bioactive coatings have the potential to reduce and eradicate the bacterial adhesion and biofilm formation of important human pathogens. Noteworthy is that RF, in particular, played a significant role in changing the surface chemical functionality, and substantially improved the biological activity of the resulted polymer. Sample fabricated at 10 W demonstrated a remarkable reduction in the number of cells, biovolume, or biofilm thickness, while there was no significant difference in the bacterial growth between samples fabricated at 50 W and control.

Geranium plasma polymer thin films showed several advantages, including cost-effectiveness, low density, good adhesion, uniform coverage, and considerable physical stability, besides significant antibacterial properties. This data recommends that the resultant polymer coatings could be efficiently integrated as antibacterial material into medical relevant devices, to mainly minimise bacterial adhesion, and consequently, substantially reduce hospital-acquired infections.

## Figures and Tables

**Table 1:** Surface tension parameters for water, diiodomethane (DIM), and glycerol used in experiments.

Solvent	Surface Tension Parameters, mJ/m^2^				
	γ	γ*^LW^*	γ*^AB^*	γ^+^	γ^−^
Water	72.8	21.8	51.0	25.5	25.5
DIM	50.8	50.8	0.0	0.0	0.0
Glycerol	64.0	34.0	30.0	3.9	57.4

**Table 2:** FTIR spectra assignments for geranium essential oil and geranium oil-based polymer.

Assignment	Group Frequency, cm^−1^	
	Precursor	Polymer
Stretching (OH)	3367	3436
Asymmetric stretching, methyl (–CH_2_)	2962	2961
Symmetric stretching, methylene (–CH_3_)	2926	2933
Asymmetric Stretching methyl (–CH_3_)	2872	2875
Stretching aldehyde (C=O)	2728	-
Stretching (C=O), aldehyde	1730	1708
Stretching (C=O) carbonyl	1713	-
Alkenyl (C=C)	1671	1625
Asymmetric bend methyl (C–H)	1452	1453
Symmetric bend methyl (C–H)	1377	1376
In-plane bending (O–H)	1267	Merged in broad band
Skeletal (C=C)	1174	
Stretching, alcohol (C–O)	1058 and 1008	

**Table 3:** Surface profiles of 3 µm × 3 µm and 10 µm × 10 µm of geranium oil-derived film surfaces fabricated at various RF power.

Sample	10 W		25 W		50 W		75 W		100 W	
Scanning area (µm)	3 × 3	10 × 10	3 × 3	10 × 10	3 × 3	10 × 10	3 × 3	10 × 10	3 × 3	10 × 10
Max, *S*_max_ (nm)	1.93	3.75	2.39	4.21	3.07	3.52	6.59	6.02	8.07	8.30
Average roughness, *S*_a_ (nm)	0.18	0.23	0.21	0.23	0.29	0.30	0.63	0.58	0.69	0.60
Root mean square, *S*_q_ (nm)	0.23	0.30	0.27	0.30	0.36	0.38	0.81	0.74	0.89	0.77
Surface skewness, *S*_sk_	0.02	0.08	0.17	0.03	0.04	0.04	0.56	0.75	0.59	0.67
Coefficient of kurtosis, *S*_ka_	0.06	0.55	0.05	0.81	0.01	0.03	0.54	1.37	0.69	1.07
Entropy	3.09	3.45	3.28	3.44	3.72	3.80	4.84	4.70	4.98	4.75

**Table 4:** Mechanical properties of geranium oil-derived thin polymer films fabricated at various power.

Power Deposition (W)	Hardness (GPa)	Elastic Modulus (GPa)	Contact Depth (nm)	Contact Stiffness (µN/nm)	Final Depth (nm)	Contact Area (nm^2^) × 10^5^
10	0.63	9.39	141.88	10.44	179.87	8.24
25	0.74	11.55	123.72	12.18	156.81	8.02
50	0.74	12.51	127.03	12.79	154.28	7.29
75	0.81	16.78	105.25	16.21	127.74	6.21
100	0.85	20.61	103.78	18.28	124.08	6.16

**Table 5:** Contact angles θ for geranium oil-derived thin polymer films deposited at varied RF powers.

Solvent	Contact Angle				
	10 W	25 W	50 W	75 W	100 W
**Water**	54.0	59.8	61.7	64.1	65.6
**DIM**	38.0	34.6	34.2	33.0	32.4
**Glycerol**	35.6	54.1	57.2	56.7	57.2

**Table 6:** Surface tension parameters and corresponding solubility for geranium oil-derived thin polymer films deposited at varied RF powers.

Sample	Surface Parameters				Interfacial Surface Tension Between Solute and Solvent			Surface/Liquid Solubility		
	γ*^LW^*	γ^+^	γ^−^	γ	γ^SL water^	γ^SL(DIM)^	γ^SL,Gycerol^	ΔG^water^	ΔG^DIM^	ΔG^Glycerol^
10 W	40.6	5.88	29.82	67.08	0.75	27.05	−1.61	−1.5	−54.10	3.22
25 W	42.21	1.21	22.06	52.54	6.134	10.72	5.48	−12.26	−21.45	−10.96
50 W	42.39	0.87	20.33	50.80	7.84	8.79	6.85	−15.68	−17.58	−13.70
75 W	42.93	1.07	17.33	51.54	10.67	8.94	6.94	−21.34	−17.88	−13.88
100 W	43.19	1.03	15.85	51.27	12.24	8.38	7.45	−24.49	−16.76	−14.90

**Table 7:** Comparative evaluation of bacterial attachment and retention on geranium oil-based polymer film surfaces fabricated at different RF powers.

Quantification	*S. aureus*	*P. aeruginosa*	*E. coli*
Initial cell density × 10^6^, cfu mm^−2^	19.6 ± 2.1	15.0 ± 0.9	9.2 ± 1.7
Zeta potential, mV	−33.1 ± 2.0	−15.1 ± 1.1	−39.5 ± 0.6
Cell dimensions, µm			
Control	0.9 × 0.5 × 0.3	2.2 × 1.2 × 0.4	2.7 × 1.2 × 0.3
10 W	0.8 × 0.4 × 0.2	1.7 × 1.1 × 0.3	2.3 × 1.1 × 0.2
50 W	0.9 × 0.6 × 0.4	2.1 × 1.1 × 0.4	2.6 × 1.4 × 0.2
Percentage of attached cells, %			
Control	0.39 ± 0.15	0.42 ± 0.11	0.49 ± 0.19
10 W	0.040 ± 0.002	0.070 ± 0.003	0.030 ± 0.001
50 W	0.33 ± 0.09	0.41 ± 0.15	0.35 ± 0.18
Retained cells 10^5^, number of cells per mm^2^			
Control	7.64 ± 1.32	6.3 ± 1.62	4.51 ± 1.29
10 W	0.78 ± 0.02	1.05 ± 0.03	0.28 ± 0.02
50 W	6.45 ± 1.45	6.15 ± 1.38	3.22 ± 1.15
Biovolume, µm^3^/µm^2^			
Control	8.92 ± 0.79	7.39 ± 0.62	8.01 ± 0.97
10 W	0.28 ± 0.03	0.23 ± 0.03	0.27 ± 0.01
50 W	7.63 ± 1.13	7.08 ± 1.02	6.03 ± 0.95
Average biofilm thickness, µm			
Control	17.19 ± 2.05	13.2 ± 1.62	11.5 ± 1.62
10 W	0.35 ± 0.03	0.42 ± 0.01	0.28 ± 0.02
50 W	15.32 ± 1.05	6.15 ± 1.38	9.22 ± 1.08
